# Degradation, Osmosis and the Emergence of Basic Functionalities in Abiotic Synthetic Life-like Chemical Systems

**DOI:** 10.3390/life16071204

**Published:** 2026-07-21

**Authors:** Chenyu Lin, Juan Pérez-Mercader

**Affiliations:** 1 Department of Earth and Planetary Sciences and Origins of Life Initiative, Harvard University, Cambridge, MA 02138, USA; chenyu_lin@fas.harvard.edu; 2 Santa Fe Institute, Santa Fe, NM 87501, USA

**Keywords:** life beyond biochemistry, synthetic life, origins of life, protocells, polymerization-induced self assembly, self reproduction, photooxidation, chemical evolution

## Abstract

Autonomous and interconnected multiscale out-of-equilibrium processes, including chemical and physical feedbacks, occur at the micron and lower scales during the heterotrophic synthesis of simple abiotic protocells, such as the ones developed in our group, with primitive life-like properties. These processes provide a solution to the autopoiesis and concentration problems in life’s origin. They also underlie the emergence of whole-vesicle functionalities like chemotaxis and self-reproduction involving dissipation and degradation which are integrated into our life-like abiotic systems. Such abiotic systems can be considered simple examples in the ontological classification of “life beyond biochemistry” (LBB), as they are based on carbon chemistry and, by design, do not use any biochemical compounds to integrate the basic system-level properties of natural life. This contribution analyzes a class of highly-out-of-equilibrium LBB systems we call “phoenix” and connects degradation pathways due to the presence of oxygenic species to enabling basic functionalities in these simple life beyond biochemistry systems such as protocell self-reproduction and the mechano-chemical squirting out into the medium of a fraction of their partially reacted lumen that enables heritable variation in our systems. We conclude that degradation within the lumen of mature micelles turning into vesicles may also be considered as a driving force for chemical evolution due to the number of new proximate reaction pathways it can open up.

## 1. Introduction

Living systems on Earth embedded in their environments and using chemistry concurrently process information, metabolize, self-reproduce, and evolve [1,2,3] following a CDC (Cell Division Cycle) (see [4] and the SI in [5]).

It is believed that at least since the Last Universal Common Ancestor of our extant biochemical life (abbreviated with the acronym LUCA) life in our planet is expressed with biochemistry. However, abstracting the notion of life to the above set of properties permits the laboratory creation of carbon chemistry but not biochemistry based simple protocells. These experimental systems also open up a far larger, and in many ways simpler and less constrained, chemical space for life than the one spanned by biochemistry (based on sugars, amino acids, nucleic acids and lipids).

During the past decade, using a combination of top-down and bottom-up approaches [5,6,7,8] our group has constructed in a one-pot batch reactor artificial, strictly biochemistry-free, chemically operated self-booting synthetic systems that also express concomitantly the above set of properties. These systems provide simple examples of the above chemical spaces: starting from non-amphiphilic abiotic molecules of very low complexity, our autopoietic carbon-based systems produce polymer amphiphiles that self-assemble to boot-up dynamical vesicular protocells capable of expressing the above properties during their “life cycle” (for example in the ones we called “Phoenix” [6]). They operate in a heterotrophic environment [4,9], which means that ab initio it contains all the necessary precursor molecular components for system generation. Surprisingly, the number of necessary ab-initio molecular species is both very small and “prima facies” quite simple. An example of this class of systems is what we call “phoenix” protocells, which once booted grow to a maximum size at which they collapse via a form of chemically controlled partial cavitation, and release to the medium partially reacted amphiphiles which then behave as a form of spores [5]. These contain sufficient conformational information to restart their process of population growth. Indeed, by adjusting the dissolved oxygen level in the aqueous medium, the system autonomously boots-up nanoscale sized self-assembled micelles which evolve into giant vesicles exhibiting primitive forms of functionality that include the above cyclic growth-implosion-rebirth episodes, phototaxis, and increases in vesicle population. From now on in this contribution, we will focus our analysis to only “phoenix” behavior.

The potentially autopoietic (self-booting) nature of the blend of initial molecules is activated and enabled by a photopolymerization reaction that ex-novo (we prefer to use this term instead of the more common term “de novo”, as in latin ex-novo implies “emergence” and not “again” as implied by the preposition “de” preceding “novo”) generates amphiphiles from the initially inert in darkness heterotrophic mixture. This photochemical polymerization process plays the role of a primitive metabolic function: it transduces the light energy into the generation of amphiphiles from simpler molecules in the medium. As the concentration in the aqueous medium of the produced amphiphiles increases, the hydrophobic effect becomes more relevant [10] and leads to their self-organization and dynamical self-assembly [11] into out of equilibrium micelles [12].

The key to their laboratory autopoiesis as dynamical protocells lies on the induction under the control of a set of intertwined physico-chemical feedbacks enabled by these chemical agents [10,13] between information and metabolism within the above heterotrophic medium. The formation of their container also impacts the internal chemical environment of the micelles. Together they provide a form of autopoiesis [3,14,15]. Indeed the minimization of the free-energy is optimized to a slow enough rate during the polymerization reaction (so that there is no product precipitation) which allows nucleation into micelles and the subsequent formation of the container that evolves from micelles to vesicles consistent with changes in the amphiphile packing parameter. (This realizes the scenario for confinement of reactants and substrates proposed by e.g., Rasmussen [16]).

A first step to elucidate the potential for molecular evolution [17,18] of these systems consists in determining which chemical and physical pathways can they follow as they degrade due to the presence in the environment of factors that impact degradation.

In what follows, we present a systematic study focusing on the understanding of the physicochemical pathways behind the observed Phoenix phenomena [6,19]. These pathways involve two oxygen-dependent photochemical reactions: photo-mediated controlled radical polymerization and photooxidation. The former induces the self-organization and self-assembly of nanoscale micelles from the starting homogeneous heterotrophic blend, while the latter combines vesicle hydrodynamics with partial chemical degradation, amphiphile packing defects, osmosis and coalescence occurring within the structurally and autonomously evolving micelles-turned-vesicles under the control of chemical forces and environment.

## 2. Analysis of Processes, Degradation and Discussion of the Phoenix Type Functional Protocells

### 2.1. Photo-Mediated Polymerization Induced Self-Assembly (PISA)

**Figure 1:**
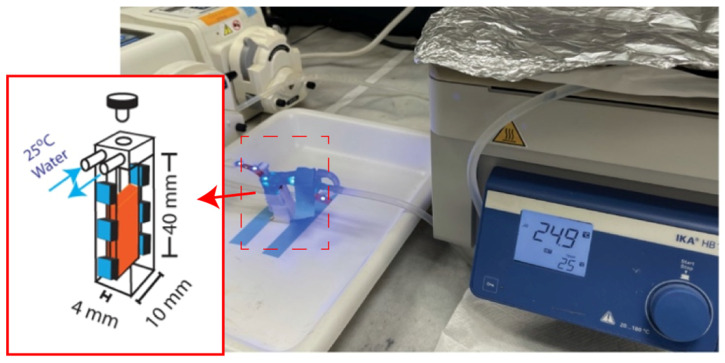
Experimental setup for our one-pot autopoietic synthetic system.

**Figure 2:**
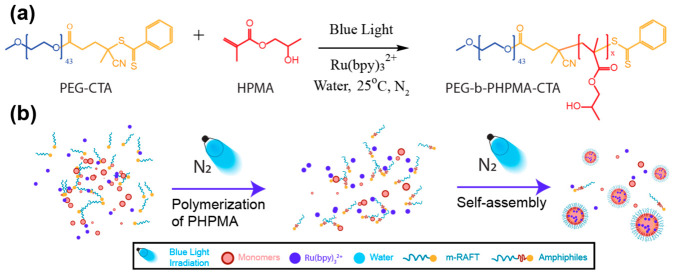
(**a**) Photo-mediated controlled radical polymerization (Adapted with permission from reference [19]), (**b**) illustration for the process of polymerization-induced self-assembly.

**Figure 3:**
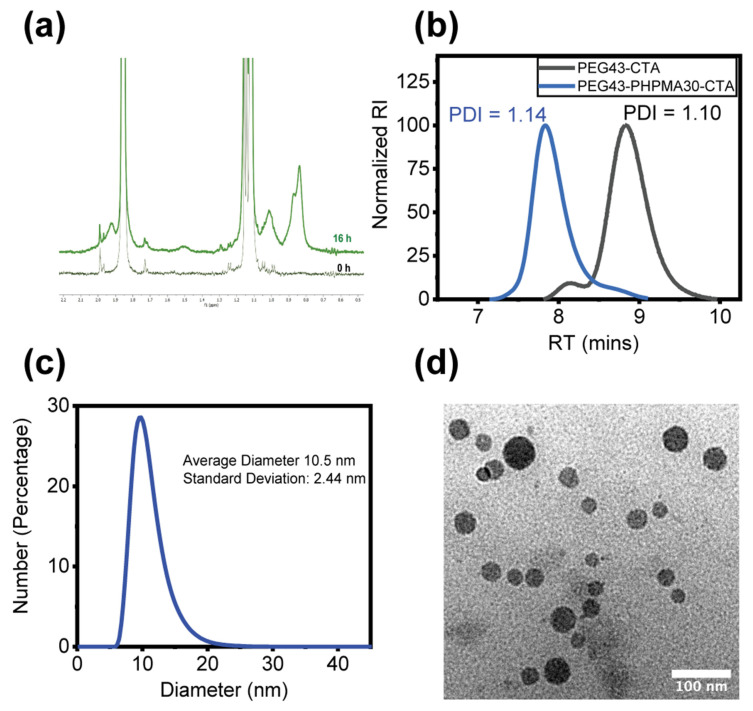
Characterization of products generated during PISA: (**a**) proton-NMR, (**b**) GPC, (**c**) DLS, (**d**) TEM (Adapted with permission from reference [19]).

The synthesis of our nanoscale micelles is achieved through a process of photo-mediated PISA operating in an initially homogenous aqueous chemical solution in a heterotrophic environment. The chemical blend contains a poly(ethylene glycol) chain transfer agent (or PEG-CTA), hydroxypropyl methacrylate monomer (HPMA), a photocatalyst molecule Tris(2,2′-bipyridyl)dichlororuthenium(II) hexahydrate (Ru(bpy)_3_^2+^), and 18 MOhm water. While in darkness, this blend is effectively inert, but allows the photo-mediated controlled radical polymerization to occur when under 470 nm light irradiation at 25 °C in an oxygen-poor environment, Figure 1. As the reaction progresses, hydrophobic PHPMA blocks are extended on the hydrophilic PEG-CTA molecules leading to formation of amphiphilic block copolymers, Figure 2a, that drive self-organization and self-assembly to form nanoscale micelles, Figure 2b. Their characterization is shown in Figure 3.

### 2.2. Oxygen Dependent Morphological Dynamics

**Figure 4:**
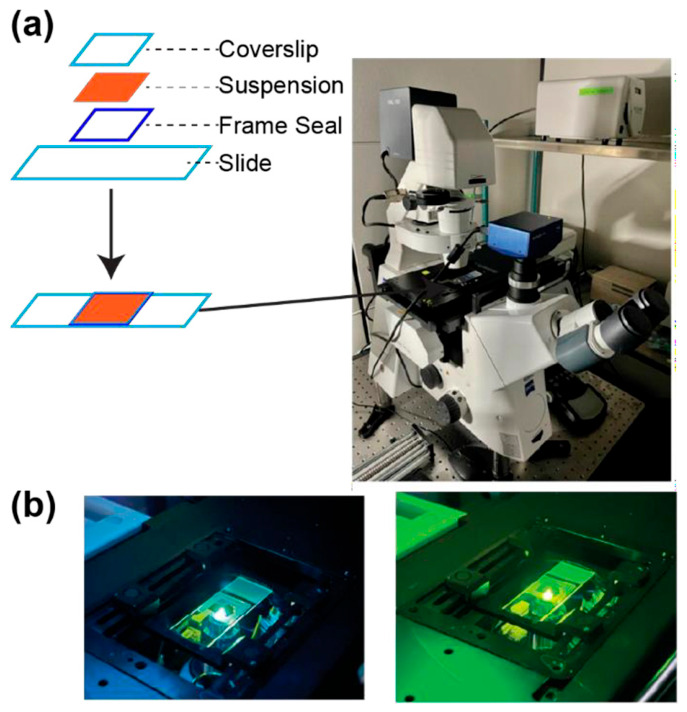
(**a**) PISA specimen preparation and (Zeiss Axiovert) inverted microscope setup, (**b**) blue light for photooxidation and green light for imaging. The system is mounted on a precisely levelled and self-isolating optical table.

To study their dynamical evolution, the as-formed nanoscale micelle suspensions [20] are mixed with rhodamine 6G dye to stain them and eventually (i.e., as they grow into micron-sized objects) observe them using fluorescence microscopy. Once stained, the system is subject to 15-min air or N2 bubbling to respectively prepare oxygen-rich or oxygen-poor PISA suspensions, which are then individually transferred to a frame-sealed glass slide for specimen preparation, Figure 4a. The time evolution of the resulting self-assembled objects in the PISA suspension is followed using an optical microscope, Figure 4a, which provides 470 nm blue photons for photochemistry and 531nm green light pulses for imaging, Figure 4b. The specimens display significantly different morphological dynamics depending on the absence or presence of dissolved oxygen.

**Figure 5:**
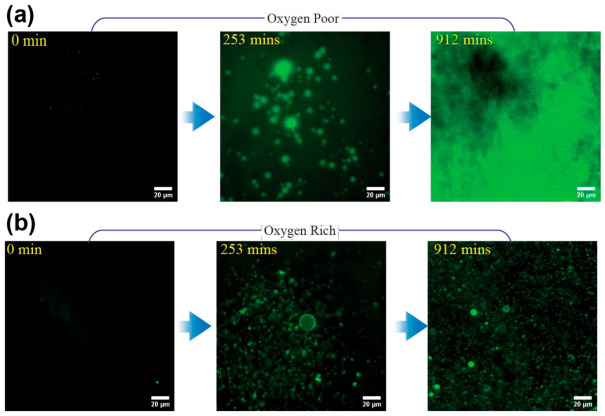
Time evolution for two morphological dynamics in PISA specimens: (**a**) gelation takes place in an oxygen-poor PISA specimen, (**b**) “Phoenix” dynamics take place in the oxygen-rich PISA specimen (Adapted with permission from reference [19]).

For the oxygen-poor PISA suspension, gelation takes place and intensifies with time as blue light irradiation continuously provides the energy to induce the swelling and “worm” (cylindrical micelles) transformations of micelles due to photo-mediated controlled radical polymerization occurring within them [21,22], Figure 5a.

In sharp contrast to the previous case, for the oxygen-rich PISA suspension microscale giant vesicles emerge, increase in number and gradually populate the entire field of view, Figure 5b.

### 2.3. Emergent Behaviors: Phoenix Dynamics, Phototaxis and Proliferation

**Figure 6:**
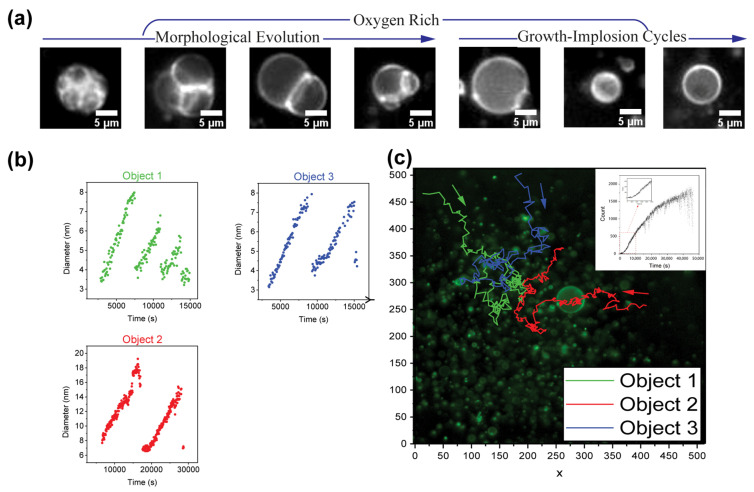
(**a**) Morphological transformation of a giant vesicle and its cyclic growth-implosion-regrowth activities. (**b**) Diameter of three individual vesicular domains over time, (**c**) Tracks of these vesicles observed in the microscope, the population growth profile (inset) and their distribution in the field of view (Adapted with permission from reference [19]).

The eventual emergence of giant vesicles from a homogeneous blend involves autonomous and chemically controlled [15,23,24,25] consecutive morphological transformations linked to water compartmentalization and formation of inverted micelles inside the self-assembled objects (cf. below). For oxygen-rich PISA, the blue light irradiation induces the formation and growth of water compartments within the hydrophobic cores of the self-organizing and self-assembled objects. The highly out of equilibrium objects thus exhibit size growth and become elongated. As more internal water compartments form and ultimately coalesce into a single lumen, the objects transform into vesicles (protocells) with a single-bilayer membrane. We call this process Stage 1 of Phoenix. With continuing growth of the water lumen, the vesicle enters a hydrodynamically-assisted stage of growth-implosion-regrowth cycles, Figure 6a, which can continue for several cycles, Figure 6b. We call this process Stage 2 of Phoenix. Meanwhile, the active vesicle population shows the emergence in the individual members of the population of light-induced Marangoni migration [6,19,24] due to which the vesicles in the field of view migrate toward the light beam center, Figure 6c [6]. As the above growth-collapse takes place nascent objects continuously emerge in the field of view and start to perform their own Phoenix cycles. This process leads to an increase in vesicle population with a single sigmoidal growth curve as shown in the upper right-hand side inset in Figure 6c.

### 2.4. Photochemistry, Hydrodynamic Evolution and Mechanisms Behind the Observed Emergent Behaviors of Our Phoenix Protocells

**Figure 7:**
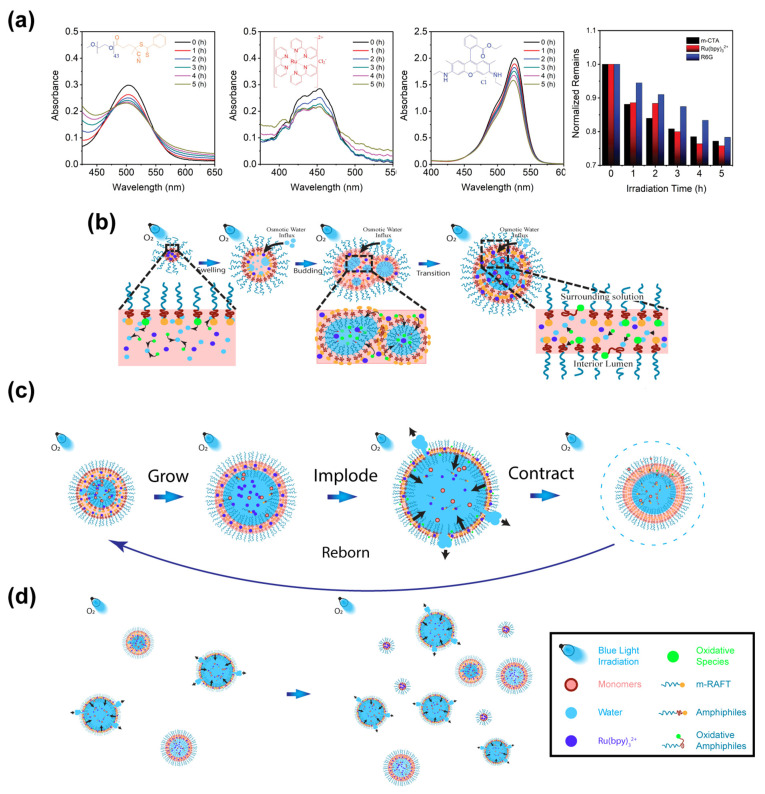
(**a**) UV-VIS spectral analysis for photo-induced chemical degradation in the presence of oxygen. From left to right: PEG-CTA, Ru(bpy)_3_^2+^, rhodamine 6G and their corresponding normalized remaining levels, the reduction in the absorption reflects the structural degradation of their chromophores. (Adapted with permission from reference [19]) Phoenix mechanism for (**b**) micelle-to-vesicle transition, (**c**) cyclic growth-implosion-rebirth episodes, and (**d**) schematic increase in vesicle population.

In Figure 7a we present the UV-VIS spectral analysis within the visible light range for characteristic absorption of three essential reagents in our PISA system: the m-RAFT agent, Ru(bpy)_3_^2+^, and rhodamine 6G. The insets in each spectrum highlight the different chromophore structures associated with the distinct absorption ranges for each of these key molecules.

The chromophore in the m-RAFT agent is the thiocarbonylthio group which allows n → π* spin-forbidden electron transition upon light absorption [26,27]. The Ru(bpy)_3_^2+^ molecule exhibits its characteristic absorption through metal to ligand charge transfer [28] and finally we see that the absorption of rhodamine 6G results from π → π* electron transition on its resonance structure [29].

Although they have different chromophore structures, all three reagents share their ability to absorb blue light (470 nm). Therefore, under blue light irradiation the reduction in intensity of such characteristic absorption in the presence of oxygen, as seen in Figure 7a, implies the occurrence of photodegradation leading to the structural degradation of the chromophores on each of these molecules [27].

The structural stability of the m-RAFT agent is strongly sensitive to irradiation with light with wavelengths within its characteristic absorption range [26]. When exposed to blue light irradiation, the m-RAFT agent undergoes photolytic fragmentation via a chain transfer mechanism [27]. The process generates two types of radicals: carbon centered radicals which can react with monomers for polymer chain extension through radical polymerization and thiocarbonylthiyl radicals with the chromophores which can bind the propagating radicals to control the extension of polymer. The decomposition of thiocarbonylthiyl radicals can take place through further photolytic cleavage and produce small molecules (e.g., CS2). This results in the loss of its blue light absorbing property and the livingness for control of polymer propagation [27,30]. In addition, with the presence of ground state oxygen, these radicals can be rapidly quenched and undergo irreversible oxidative reactions due to their faster reaction rate with oxygen than with monomers. The oxidative reactions not only stop polymer propagation but also produce hydroperoxide (or ROOH) [31]. The hydroperoxide molecule can further degrade to generate alkoxy radicals (or RO•) which are involved in several chemical pathways for production of oxidized products including ketone, aldehyde, ester and alcohol leading to the subsequent structural degradation of the chromophore of the m-RAFT agent and the erosion of its living fidelity in mediating the radical polymerization process [32,33,34].

The other two molecules, Ru(bpy)_3_^2+^, and rhodamine 6G are well-known photocatalysts which can accelerate the progress of RAFT radical polymerization [6,35,36]. They also experience structural degradation and attenuation of their light absorption under irradiation. Unlike the m-RAFT agent that reacts with ground state oxygen through generated radical species, the Ru(bpy)_3_^2+^ and rhodamine 6G molecules undergo degradation through excited oxygen (or singlet oxygen) due to their photosensitization properties [37,38]. When exposed to both light and ground state oxygen, these photocatalysts undergo electron excitation upon light absorption which is then followed by intersystem crossing to a triplet excited state [39]. In this situation, the longer triplet excitation lifetime allows the energy to be efficiently transferred to nearby ground state oxygen resulting in production of singlet oxygen [39,40]. Additionally, singlet oxygen is believed to cause photobleaching of the photocatalysts as it is a highly reactive electrophile capable of rapidly reacting with unsaturated carbon-carbon bonds or neutral nucleophiles including sulfide and amines [37,40]. As a result, irreversible oxidations take place at those unsaturated chemical structures, like the bipyridine ligand of ruthenium complex or the aromatic rings of rhodamine 6G. Such structural degradation decreases their light absorption capability and, consequently, reduces their contribution to the polymerization progress.

Our synthetic system contains micelles which are autonomously booted up in a heterotrophic environment through the mechanism of polymerization induced self-assembly under blue light irradiation. When the system is exposed to air, both ground state oxygen and photosensitized singlet oxygen can affect polymerization reactions and contribute to structural degradation of the building blocks and the molecules entrapped within the cores of these micelles during micelle self-assembly [41]. This significantly impacts the dynamics and the sequence of morphological time-evolution of the micelles. In an oxygen rich environment the ground state oxygen will diminish the rate of on-going radical polymerization and remove the end group of the m-RAFT molecules by reacting with the propagating radicals to generate peroxy species [32,42,43]. Furthermore, the singlet oxygen induced by the photosensitization process of Ru(bpy)_3_^2+^ and rhodamine 6G can act as a cofactor for structural degradation by forming hydroperoxides through irreversible oxidative reactions at the unsaturated bonds ubiquitously existing within the micelle cores [37,39]. The resulting peroxy groups from both photochemical pathways promote breakdown of molecules including Ru(bpy)_3_^2+^ and rhodamine 6G across the micelle cores and produce water soluble oxidized products [32,44,45,46,47].

Such chemical degradation limits the chain extension of hydrophobic blocks and prevents the associated formation of gelation. Moreover, the packing parameters of the amphiphiles in micelles which contain diverse building blocks (e.g., the hydrophobic blocks with limited chain extension and oxidized hydrophobic blocks) will exhibit significant alteration in the local packing amphiphiles. This not only creates packing defects with increased hydrophilicity in the hydrophobic back bonds but enhances the water permeability within the micelles [46,47]. The small water soluble oxidized products generated during the molecular degradation can diffuse into proximate water domains in a hydrated micelle core and induce an osmotic pressure mismatch with the external medium, Figure 7b [48]. Coupled with the enhanced water permeability, the osmotic imbalance will drive water influx from the surrounding medium, and create expansion pressure, which results in the formation in the interior of the micelle of small inverted micelles that gradually grow in size and eventually coalesce among themselves to form the cylindrical micelle cores, Figure 7b.

With the progress of photooxidation-induced structural degradation, inverted micelles continuously form internally, grow and eventually coalesce into a single lumen, accompanied with the expansion in size and rapid reconfiguration of the packing amphiphiles to form single bilayer membranes, Figure 7b.

At this point we recall that the acceleration of the membrane dynamics of a vesicle immersed in a solution results from the simultaneous action of osmotic gradients, membrane stretching, and changes in the membrane curvature as the vesicle expands (this dynamics is incorporated and integrated by the Rayleigh-Plesset equation [49] which is the truncated form of the Navier-Stokes equation for the membrane of a vesicle in a fluid). For some parameter values characterizing the latter, the solutions to this equation display cyclic bubble (vesicle) cavitation-growth episodes. These complex dynamics can indeed occur in our vesicles as a consequence of multiple feedbacks between the chemical forces associated with polymerization, degradation, and osmotic gradients as we described in the previous paragraphs and is seen in our experiments.

Indeed, as the photooxidation continues, the growing water lumen “vesicle originating from a dynamical micelle” can reach a critical volume where the membrane becomes unstable and ruptures to relax the expansion-creating pressure and, consequently, release to the medium a fraction of their internal and constituent materials [50], Figure 7c. That is, the relaxation results in the membrane elastic energy pulling together some areas in the vesicle membrane which then collapses the membrane, allowing it to inelastically contract and restore enough of its integrity for a subsequent growth episode of the initial (parent) vesicle. This has Important consequences for the dynamical evolution of the vesicular protocells: as the cavitation occurs a substantial fraction of the internal (partially reacted and chemically active or “living”) amphiphiles are “squirted” into their external heterotrophic medium.

The released materials during the cavitation event cause the micelle-vesicle building block concentrations in the regions of the heterotrophic bulk adjacent to the collapsing vesicle, to quickly go above the critical micelle concentration, thus driving the formation of new self-organizing amphiphiles and their self-assembled objects using “food” found in the medium.

Effectively, the above squirted amphiphiles act like “spores” (or “sporules”) from which new micelles and vesicles eventually emerge and their non-linear population growth manifests as a form of self-reproduction. Because of the way they are generated from their parents, the newly produced objects have similar (but not necessarily identical due to small variation generated by the polydispersity of their “living” and degrading hydrophobic tails) components to their parents which, so long as there is enough supply of materials in the reactor (i.e., their environment), will also grow and implode to release materials through photooxidation during several juxtaposed cycles of collapse and resuscitation.

As an important consequence, the vesicle population increases non-linearly, Figure 7d.

## 3. Conclusions

We have explored the chemical foundations for the chemomechanical behaviors taking place in abiotic and photochemistry controlled vesicular systems self-booting in a heterotrophic environment undergoing RAFT PISA (Polymerization Induced Self-Assembly). These behaviors include photo-chemotaxis and self-reproduction during consecutive out-of-equilibrium cycles involving internal processes in the self-assembled supramolecular objects coupled to the external environment via several interconnected chemical and mechanical feedbacks.

Chemical degradation affects molecular species in a living polymerization reaction within vesicles, contributing to metabolism-like process within the vesicle lumen.

We found that the presence of oxygen, which promotes degradation and can affect the polymerization reaction, determines which route the system’s collective morphological evolution will follow. When poor in oxygen, gelation dominates. Conversely, in the presence of abundant oxygen, photooxidation dominates, leading to chemical degradation and osmotic imbalance. This triggers multiple feedback mechanisms between the chemistry, membrane composition, and mechanical properties of the vesicle, which are regulated by the relative significance acquired in this process by the terms in the Rayleigh-Plesset equation for a membrane [49]. The oxidative physicochemical pathway sustains the system’s out-of-equilibrium dynamical state and facilitates the emergence of giant vesicles, from initially active micelles, which then go on to execute autonomous cyclic growth-collapse episodes accompanied by a range of life-like activities, including photo-chemotaxis and apparent self-replication-like population growth.

We end by commenting briefly on the direction to which our findings point: chemical evolution. Specifically, chemical degradation can contribute to the generation of chemical diversity within the lumen by producing chemically proximate species. This leads to the subsequent opening of internal competition among existing and new chemical species and reaction pathways, becoming a source of chemical change and thereby serving as a catalyst for chemical transformation. Furthermore, internal competition can serve as a catalyst for viable chemical evolution in such systems. If the induced molecular innovations are chemically close enough to be adopted and transmitted to subsequent generations of vesicles produced through self-reproduction, degradation can act as a promoter of chemical evolution. The ability to “drink” material from the medium presents a scenario for these abiotic systems to assimilate some of the novel chemical species in the medium resulting from its own evolution and generate additional opportunities for the co-evolution of medium and abiotic systems [4,9].

Finally, the above delineates a pathway towards the emergence of functions in the context of life beyond biochemistry (LBB), as environment and ecology combine with the unavoidable degradation and chemical evolution to use chemically controlled osmotic forces as the enablers of function which make possible system persistence.

## Data Availability

No new data were created or analyzed in this study. Data sharing is not applicable to this article.
